# Volatile organic compounds (VOCs) from *Bacillus subtilis* CF-3 reduce anthracnose and elicit active defense responses in harvested litchi fruits

**DOI:** 10.1186/s13568-019-0841-2

**Published:** 2019-07-27

**Authors:** Pengyu Zhao, Peizhong Li, Shiyuan Wu, Minshun Zhou, Ruicong Zhi, Haiyan Gao

**Affiliations:** 1 School of Life Sciences, Shanghai University, Shanghai, 200444 People’s Republic of China; 2 School of Computer and Communication Engineering, University of Science and Technology Beijing, Beijing, 100083 People’s Republic of China

**Keywords:** *B. subtilis* CF-3 VOCs, *C. gloeosporioides*, Antibacterial effect, Litchi fruit

## Abstract

In this study, we investigated the effects of volatile organic compounds (VOCs) produced by *Bacillus subtilis* CF-3 on the growth and development of *Colletotrichum gloeosporioides* and evaluated the elicitation of active defense responses in harvested litchi fruits. In vitro experiments were conducted to explore the bacteriostatic effect of VOCs in inhibiting pathogenic fungi by means of plate enthalpy test, scanning electron microscopy, transmission electron microscopy, and gas chromatography–mass spectrometry (GC–MS). The results showed that 2,4-di-*tert*-butylphenol and CF-3 24-h fermentation broth (24hFB) can significantly inhibit the germination of fungal spores, disrupt hyphal and cell morphology, and decrease cell membrane fluidity and integrity, resulting in the changes of indexes. In addition, the bacteriostasis of VOCs in the defensive ability of litchi fruits to *C. gloeosporioides* was studied, and it was shown that 2,4-di-*tert*-butylphenol and CF-3 24hFB can inhibit the activity of the pathogenic enzymes (pectinase and cellulase) secreted by *C. gloeosporioides* to reduce the decomposition of plant tissues, activate antioxidant enzymes (peroxidase, polyphenol oxidase, catalase, and superoxide dismutase) in the fruit to eliminate excessive reactive oxygen species in fruits in order to reduce plant cell damage and activate disease resistance enzymes (phenylalanineammonialyase, chitinases, β-1,3-glucanase) to enhance the resistance of litchi fruits to *C. gloeosporioides* and inhibit its growth. This study investigated the bacteriostasis of VOCs in inhibiting *C. gloeosporioides* and inducing the resistance of litchi fruits, providing a theoretical basis for future applications.

## Introduction

Litchi anthracnose is a common disease of litchi that mainly affects young leaves, flower spikes, and mature or close to mature fruits, resulting in rotten flower and fruit (Liu 2006). For a long time, one or a combination of several methods, such as refrigerated atmosphere storage, chemical agents, physical treatment, etc., have been used to keep litchi fresh and disease-free after harvesting (Wang et al. 2001). In addition to antioxidants and food additives, the main components of litchi antiseptic preservatives are chemical fungicides, including organic and inorganic compounds, such as prochloraz, sorbate, NiCl_2_, etc., which have different degrees of toxicity and residual after use (Qi et al. 2015). Therefore, it is particularly important to seek safe, nontoxic, and efficient technology for controlling litchi post-harvest decay to promote the healthy development of litchi industry. Therefore, the current hotspot of biological control, as well as the efficient and safe solution for litchi fruit decay, is to screen beneficial microorganisms with antagonistic action and to extract relevant antibacterial chemicals to inhibit or kill pathogenic microorganisms in order to prolong the storage period and improve fruit preservation.

In recent years, the antibacterial effect of *B. subtilis* has been a research topic of interest worldwide. *B. subtilis* is a mesophilic aerobic or facultative anaerobic gram-positive rod-shaped bacterium (Sonenshein and Losick 1994). It is widely distributed in nature, nontoxic and harmless to humans, easy to isolate and cultivate, and has broad-spectrum antibacterial activity. The *B. subtilis* vegetative cells are affected by changes in external environmental factors, such as nutrient deficiencies, accumulation of metabolites, temperature changes, etc. Endospores, which are highly resistant to radiation, heat drying, extreme pressure, hydrostatic pressure, and some toxic chemicals (Nicholson et al. 2000; Setlow 2010), are formed intracellularly and have strong anti-reverse ability to produce antibiotics and enzymes, peptides, lipopeptides, polyenes, amino acids, etc., (Ahimou and Deleu 1999). Waewthongrak et al. (2015) showed that the cyclolipopeptides secreted by the *B. subtilis* strain ABS-S14 elicit a broad-spectrum antibiotic effect, reducing the number of *Penicillium* strains exposed to *B. subtilis* and cell-free cultivation by 96.2% and 90.9%, respectively. Ambrico and Trupo (2017) further confirmed that Iturin A produced by *B. subtilis* ET-1 can effectively inhibit the occurrence of blue mold on lemon fruit and gray mold on strawberry fruit. Ahmad et al. (2017) isolated and identified a *B. subtilis* strain 330-2 from rapeseed; this strain was found to secrete indole-3-acetic acid, iron carrier, lysozyme, organic and inorganic phosphates from different sources, and zinc ions. It was reported to effectively inhibit the growth of *Rhizoctonia solani* AG1-IA, *Fusarium oxysporum*, *Alternaria tenuis* Nees, *Cochliobolus heterostrophus*, and *Nigrospora oryzae*.

In the study of VOCs in *B. subtilis*, (Vespermann et al. 2007) reported the VOCs produced by seven strains of *B. subtilis* B2 against a phytopathogen by measuring the VOCs of nine different bacterial strains against the mycelial growth inhibition of *Rhizoctonia solani*. Leelasuphakul et al. (2008) isolated 205 strains of *Bacillus* from soil and selected 23 strains of *B. subtilis*. It was found that the VOCs produced by them could inhibit the growth of *Penicillium digitatum* by approximately 30–70%. Li et al. (2015) found that *B. subtilis* XF-1 and 168 can effectively inhibit the growth of *Fusarium solani*, and the main VOCs detected by solid-phase microextraction gas chromatography–mass spectrometry (SPME–GC–MS) included 2-methylbutanone, 2-pentanone, 3-methyl-2-pentanone, 2,5-dimethylpyrazine, benzaldehyde, and 5-methyl-2-heptanone. It can be seen that *B. subtilis* produces a variety of VOCs, with different VOCs having different antibacterial activities against different pathogenic bacteria. The VOCs of *B. subtilis* are composed of a variety of chemical substances, which have strong antibacterial effects and can inhibit the growth of different kinds of pathogens. However, *B. subtilis* has not been widely used in actual preservation technology because the functional effects of the main components are lack of scientific basis and volatile matter separation technology. The mechanism of the antibacterial action of related volatile substances is not proven. Therefore, the future studies should identify and explore VOCs and elucidate the mechanism of their bacteriostatic action.

Compared with non-volatile antibacterial substances, VOCs exist in the form of gas and are more easily adsorbed by plants in the form of chemical signals, thus exerting its bacteriostatic effect (Kesselmeier et al. 2002). Looking forward to the application prospect of VOCs, it is also possible to control the addition of VOCs produced by *B. subtilis* as a protective gas and to combine the existing technologies, such as changing atmosphere preservation and film closed gas modulation, for efficient preservation. In the early stage, the fermented bean curd was used to isolate a biocontrol fungus, which has good control effect on the postharvest preservation of litchi. The strain was identified as *B. subtilis*, its number is CF-3, registered and preserved at the China Center for Type Culture Collection, with the deposit number CCTCC M 2016125. In the previous study, we demonstrated that the antibacterial protein and volatile substances produced by the biocontrol bacteria have good antibacterial effects against various plant pathogenic bacteria (Gao et al. 2016). The VOCs produced by *B. subtilis* CF-3 during fermentation were isolated and identified by headspace SPME–GC–MS (HS–SPME–GC–MS). A single antibacterial activity test was carried out on 74 compounds contained in *B. subtilis* CF3 showed that 2,4-ditertiary butylphenol produced the highest inhibitory effect on *C. gloeosporioides* (Gao et al. 2017). However, the mechanism by which the volatile substances produced by *B. subtilis* inhibit the growth of *C. gloeosporioides* is not clear. Therefore, based on the results of previous investigations, this study further explored the mechanism of action of *B. subtilis* CF-3 VOCs against the anthracnose of litchi. Physical experiments were conducted to explore the mechanism of VOCs-induced defensive reaction in litchi fruits after harvest. Therefore, it provides an effective basis for the production of biological preservatives for effectively controlling *C. gloeosporioides* and, thus, for updating the biological preservation technology.

## Materials and methods

### Selection of strain

#### *Bacillus* strain

*Bacillus subtilis* CF-3 (registered in the China Center for Type Culture Collection, CCTCC M 2016125) was isolated from fermented bean curd and identified by the Laboratory of Food Safety and Quality Control (School of Life Sciences, Shanghai University) (Gao et al. 2016). *B. subtilis* CF-3 was cultivated for 7 days at 37 °C, using LB solid medium (Gao et al. 2017).

#### *C. gloeosporioides*

*Colletotrichum gloeosporioides* Penz. (registered in Agricultural Culture Collection of China, ACCC 36351, provided by the Institute of Environment and Plant Protection, Chinese Academy of Tropical Agricultural Sciences) was cultivated for 7 days at 25 °C, using the potato dextrose agar (PDA) solid medium (Gao et al. 2017).

### Fruits

Litchi fruits of the variety “Guangxi rice litchi” were purchased from Shanghai Wholesale Fruit Market. The fruits that were disease-free, had no obvious wounds, had a neat appearance, and had the same maturity and size were chosen. The selected fruits were pruned and immersed in a 0.1% sodium hypochlorite solution for 1 min, and then rinsed with tap water and air-dried in the fume hood for use.

### Preparation of 24-h fermentation broth from *B. subtilis* CF-3

*Bacillus subtilis* CF-3 was cultivated on LB solid medium for 24 h at 37 °C, gently scraped with an inoculating loop and transferred into 100 mL LB liquid medium in a conical flask and cultured at 37 °C in a rotary shaker at 150 r/min for 24 h. Subsequently, the culture solution was diluted to a concentration of approximately 10^8^ cfu/mL, using a hemocytometer, to obtain a seed cultivation solution and carry out a volatile bacteriostatic test.

### Effects of VOCs on *C. gloeosporioides* in litchi fruit

The disinfected and air-dried litchi fruits were subcutaneously injected at a site 5 mm deep into the pulp with 20 μL of the prepared fungal spore suspension at a point of the equator of the litchi fruits using a 1-mL syringe. The injected litchi fruits were dried in the fume hood until use. Twenty litchis were placed on a plastic tray sterilized with 75% ethanol (4 rows × 3 rows of grooves), positioning two litchis in each groove and then placing a piece of filter paper in each of the head and tail grooves (90 mm diameter) in the middle of the tray. Subsequently, 200 μL of 1 mol/L 2,4-di-*tert*-butylphenol (diluted with dimethyl sulfoxide) or CF-3 24hFB was added to each of the two filter paper sheets. Then, quickly put the tray containing litchi fruits into a cardboard box (45 cm × 35 cm × 10 cm) and sealed. The box was wrapped in a gas-conditioned bag, and kept at room temperature (25 °C). The samples were taken by unpacking the bag every day starting from d1 for a total of 4 days, with three replicates in each treatment. Owing to the instability of VOCs, the bags were discarded directly after unpacking and were not re-sealed.

$$ {\text{A}}\left( \% \right) = \frac{\Delta - \delta }{\Delta } \times 100\% $$

where A is the inhibition rate; Δ is the diameter of the lesion in the blank group; δ is the diameter of the lesion in the treatment group.

The litchi fruit treatment settings were as follows: (1) blank control group (no treatment); (2) 1 mol/L 2,4-di-*tert*-butylphenol (diluted with dimethyl sulfoxide); (3) CF-3 24 h fermentation broth.

### Effects of VOCs on *C. gloeosporioides* spore germination

Five milliliters of sterile water containing 0.05% Tween 80 was added to the PDA plate of *C. gloeosporioides* pre-cultivated for 7 days. The resulting spore suspension was then filtered through eight layers of sterile cheesecloth, and its concentration was adjusted to 1 × 10^4^ conidia/mL using distilled water.

The treatment solution was evenly spread on LB solid medium, and the spore suspension (20 μL) was evenly spread on fresh PDA medium. The reagent-coated solid medium was inverted on the PDA coated with spore suspension, and then the plate was sealed with parafilm and incubated at 28 °C. At the same time, the LB solid medium coated with the same volume of distilled water or dimethyl sulfoxide was used as a blank control, and the same volume of thymol solution was applied as a positive control and cultivated under the same conditions. Three parallel replicates were set up for each processing group. The germination of spores was respectively observed by light microscopy at 2, 4, 6, 8, 10, and 12 h, and the length of the germ tube exceeded half of the maximum diameter of spores was considered as the spore germination standard. When the spore germination rate in the blank control group exceeded 90%, the spore germination rates of all treatment groups were measured and calculated using the following formula:

1 $$ R\left( \% \right) = \frac{\text{n}}{t} \times 100\% $$

where R is the spore germination rate; n is the number of spores that have sprouted; t is the total number of spores.

The treatment settings were as follows: (1) blank control group (dimethyl sulfoxide, distilled water); (2) positive control group (500 mg/L thymol solution with distilled water); (3) 1 mol/L 2,4-di-*tert*-butylphenol (diluted with dimethyl sulfoxide); (4) CF-3 24 h fermentation broth.

### Preparation of fumigation treatment *C. gloeosporioides* by *B. subtilis* CF-3 VOCs

The method described by Arrebola et al. (2010) and Jiang et al. (2014) was used to evaluate the inhibitory effects of VOCs produced by *B. subtilis* CF-3 against *C. gloeosporioides*. After 24 h fermentation, 20 μL of *B. subtilis* CF-3 fermentation broth was aspirated, and evenly spread on LB solid medium. Subsequently, a plug (Ø7 mm) from the fungal agar, which was incubated for 7 days, was punched and placed at the center of a fresh PDA medium. Finally, the LB solid medium was inverted on the fungus-attached PDA solid medium and sealed with a parafilm to reduce the loss of VOCs. The sealed petri dish was placed in an electrothermal incubator at 28 °C for 7 days.

The treatment settings were as follows: (1) blank control group (dimethyl sulfoxide, distilled water); (2) 1 mol/L 2,4-di-*tert*-butylphenol (diluted with dimethyl sulfoxide); (3) CF-3 24 h fermentation broth.

### Effects of VOCs on the cellular morphology of *C. gloeosporioides*

Preparation of electron microscope fixative (2.5% neutral glutaraldehyde): 10 mL glutaraldehyde solution (25%), 50 mL phosphate buffer solution (0.2 M; 0.588 g NaH_2_PO_4_·2H_2_O, 5.8 g Na_2_HPO_4_·12H_2_O were dissolved and mixed in 100 mL sterile distilled water and the pH was adjusted to 7.2–7.4, standby), 40 mL sterile distilled water were mixed, and a 2.5% neutral glutaraldehyde solution was prepared.

The scraped hyphae (blank control, single VOC treatment, CF-3 24 h fermentation broth) were soaked in the electron microscope fixative and sent to Shanghai Yuyi Testing Center for electron microscopy.

### Effect of VOCs on fatty acid content in *C. gloeosporioides* cell membrane

#### Cell membrane lipid extraction Soxhlet

In a round bottom flask, 0.2 g of the fumigated fungal sample was mixed with 100 mL of methanol to reflux for 2 h, and the reflux was steamed to 5 mL with a rotary evaporator.

#### Fatty acid derivatization

The extracted lipid concentrate was hydrolyzed with potassium hydroxide-methanol solution (11 g/L) at 90 °C for 10 min. Total fatty acids were then derivatized with 2 mL sulfuric acid methanol (10%, V/V) at 90 °C for 20 min. After adding 250 μL of the internal standard (1 g/L methyl nonanoate), the fatty acid solution was extracted with 2 × 6 mL of isooctane and occasionally oscillated. The water in the isooctane layer was removed with anhydrous sodium sulfate. Finally, it was concentrated by rotary evaporation to 1 mL and stored at 20 °C for analysis.

#### GC–MS analysis

According to the method described by Tang et al. (2011), a 1 μL sample was injected in an HP-5 ms capillary column to analyze the fatty acid content though GC–MS. All samples were analyzed in triplicate, and the standard deviation was calculated.

### Effects of VOCs on the content of ergosterol in the *C. gloeosporioides* cell wall

#### Extraction and derivatization of ergosterol from the cell wall

The sample was accurately weighed to 0.2 g and placed in a 40-mL covered vial. Subsequently, 20 mL NaOH solution (5 g NaOH + 5 mL sterile distilled water + 95 mL methanol) and 20 μL of 1 mg/mL internal standard stock solution [100 mg accurately weighed 7-dehydrocholesterol standard (VD_3_) was accurated to 0.1 mg and dissolved in methanol and diluted to 100 mL to obtain 1 mg/mL internal standard stock solution]. The stock solution was hydrolyzed at 80 °C for 2 h and then cooled to room temperature (25 °C). Five milliliters of the above hydrolyzate was taken in a 15-mL stoppered tube 3 mL of *n*-hexane was added, followed by vortexing for 2 min. After standing, 1 mL of the supernatant was taken into a chromatography bottle, and the solvent was dried in a 40 °C water bath, followed by the addition of 300 μL *N*,*O*-bis(trimethylsilyl)trifuoroacetamide (BSTFA) to derivatize ergosterol at 80 °C for 45 min. After cooling, the ergosterol sample was analyzed by GC–MS.

#### GC–MS analysis

According to the method of Saraf et al. (1997), Pasanen et al. (1999), Sha et al. (2008), and Liu et al. (2007), select-ion monitoring (SIM), target ion (M/Z): ergosterol 363, and internal standard 351 were used to detect the content of ergosterol. All samples were analyzed in triplicate, and the standard deviation was calculated.

Preparation of ergosterol standard solution and preparation of standard curve were referred to Yuan et al. (2007). The standard series of ergosterol solutions were determined and analyzed by GC–MS under the same conditions. A regression analysis was used to obtain the integrated peak areas of ergosterol and internal standard.

### Effects of CF-3 VOCs on related enzyme activities and indicators in litchi fruits

The methods of selection and pretreatment of litchi varieties were the same as the methods of bacteriostasis determination. The litchi fruit treatment settings were as follows: (1) blank control group (no treatment); (2) 1 mol/L 2,4-di-*tert*-butylphenol (diluted with dimethyl sulfoxide); (3) CF-3 24 h fermentation broth.

#### Litchi fruit sampling

Ten litchis were taken from a box as a parallel. Thus, a total of 30 different litchis were taken from three boxes. The peel and the nucleus of the sampled litchis were removed and their pulps were torn into small pieces, which were frozen in liquid nitrogen and stored in a refrigerator at − 80 °C for later use.

### Measurement of polygalacturonase and cellulose

To extract polygalacturonase (PG) and cellulose, 10.0 g of litchi fruit sample was ground in 95% ethanol in an ice bath and centrifuged at 4 °C and 12,000×*g* for 20 min. The above step was repeated with 80% ethanol. The extraction buffer solution was added to the precipitate. After centrifugation, the supernatant was collected and stored at 4 °C.

The PG (or cellulose) extract (0.5 mL) was mixed with the reaction solution, and incubated at 37 °C for 1 h. Subsequently, 1.5 mL 3,5-dinitrosalicylic acid reagent was immediately added dropwise, heated in a boiling water bath for 5 min, diluted to 25 mL, and shaken. The absorbance was determined at 540 nm. The enzyme extract boiled for 5 min was used in the blank control group.

### Measurement of antioxidant system and phenylalanine ammonia-lyase

To extract peroxidase (POD), polyphenol oxidase (PPO), catalase (CAT), superoxide dismutase (SOD), superoxide anion (O_2_^−^), hydrogen peroxide (H_2_O_2_), malondialdehyde (MDA), and phenylalanine ammonia-lyase (PAL), 5.0 g of litchi fruit samples were ground in the extraction buffer solution in an ice bath and centrifuged at 4 °C and 12,000×*g* for 30 min (20 min for O_2_^−^, H_2_O_2_, and MDA). The supernatants were used as enzyme extracts.

The determination methods of POD, PPO, CAT, SOD, H_2_O_2_, O^2−^ and PAL were referred to Wang et al. (2013). The absorbance was determined at 470 nm for POD, 420 nm for PPO, 560 nm for SOD, 530 nm for O^2−^, 412 nm for H_2_O_2_, 290 nm for PAL and 240 nm for CAT, taking distilled water as a reference. The content of MDA was determined by thiobarbituric acid colorimetry. The absorbance was measured at 450, 532, and 600 nm. In the blank control group, the supernatant was replaced with 2.0 mL of tricarboxylic acid (TCA).

### Measurement of superoxide anion, chitinase, and β-1,3-glucanase

The production rate of superoxide anion and the activities of chitinase (CHI) and β-1,3-glucanase (GLU) were measured using commercial kits (Comin Biotechnology, Co., Ltd, Suzhou, CHN).

### Statistical analysis

The SPSS 18.0 software (SPSS Inc., Chicago, IL, USA) was used to conduct the statistical analysis. Duncan’s post hoc test was applied to compare the mean values. A significance level of *P *<* 0.05* was used to determine the significant differences. Mean values with standard deviations were reported. Additionally, MS Excel 2013 (Microsoft Corp., Redmond, WA, USA) was used to calculate and draw standard curves, and Origin Pro 9 (OriginLab Corp., Northampton, MA, USA) was used to create charts.

## Result

### Effects of VOCs on *C. gloeosporioides* in vivo and in vitro

**Fig. 1:**
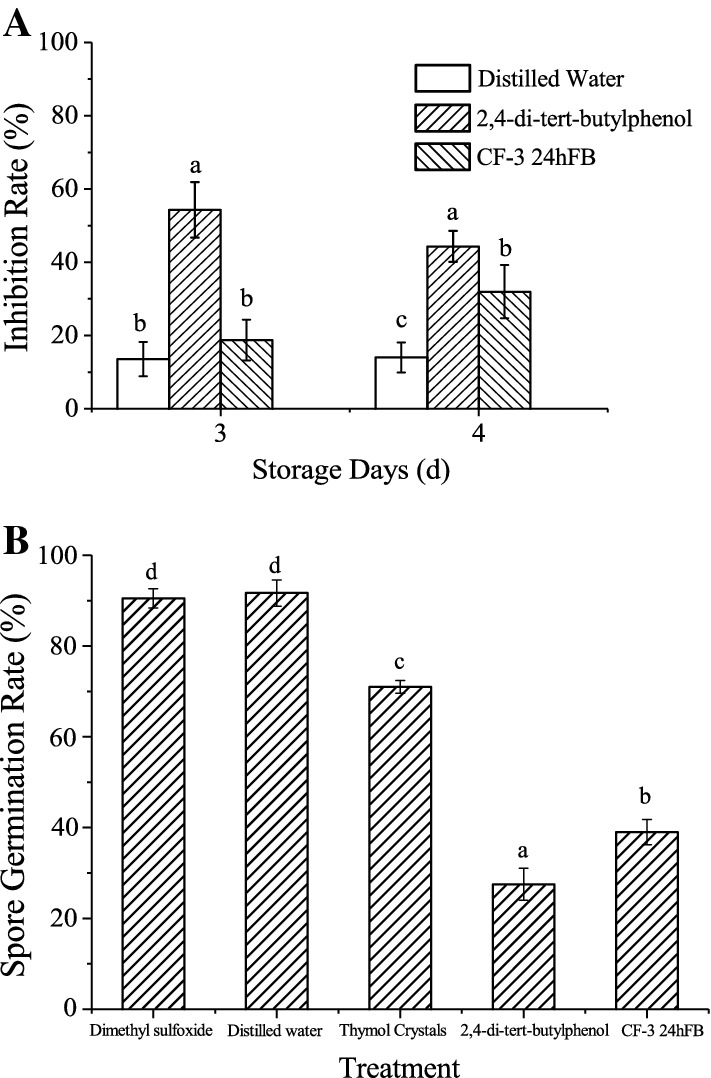
Inhibitory effect of different treatments on *C. gloeosporioides* (**A**) and spores’ germination (**B**) of *C. gloeosporioides.* Each column represents the mean value from three independent experiments, and vertical bars represent the standard errors of the means for each treatment. Different letters represent significant differences (*P *<* 0.05*)

As shown in Fig. 1A, the brown lesions appear around the injection site on d3. The inhibition rate of the 2,4-di-*tert*-butylphenol treatment group was significantly higher than the blank group (*P *<* 0.05*). On d4, the inhibition rates of the 2,4-di-*tert*-butylphenol and CF-3 VOCs treatment groups were significantly higher than the blank group (*P *<* 0.05*), indicating that the 2,4-di-*tert*-butylphenol and CF-3 VOCs can effectively inhibit the growth and development of *C. gloeosporioides* in litchi fruits.

In the spore germination test, the spores in the VOC treatment groups, as well as in the distilled water and dimethyl sulfoxide groups (blank control groups), germinated after 6 h; therefore, the spore germination rates of all groups were measured at 6 h (Fig. 1B). It could be seen from the figure that 2,4-di-*tert*-butylphenol and CF-3 24hFB could greatly inhibit the germination of *C. gloeosporioides* spores. The germination rates of the spores treated with 2,4-di-*tert*-butylphenol and 24hFB were 27.50 ± 3.53% and 39.23 ± 2.83%, respectively, indicating that the two reagent treatments could greatly inhibit the germination of *C. gloeosporioides* spores, thus playing an effective role in disease prevention.

### Effects of VOCs on the mycelial morphology of *C. gloeosporioides*

**Fig. 2:**
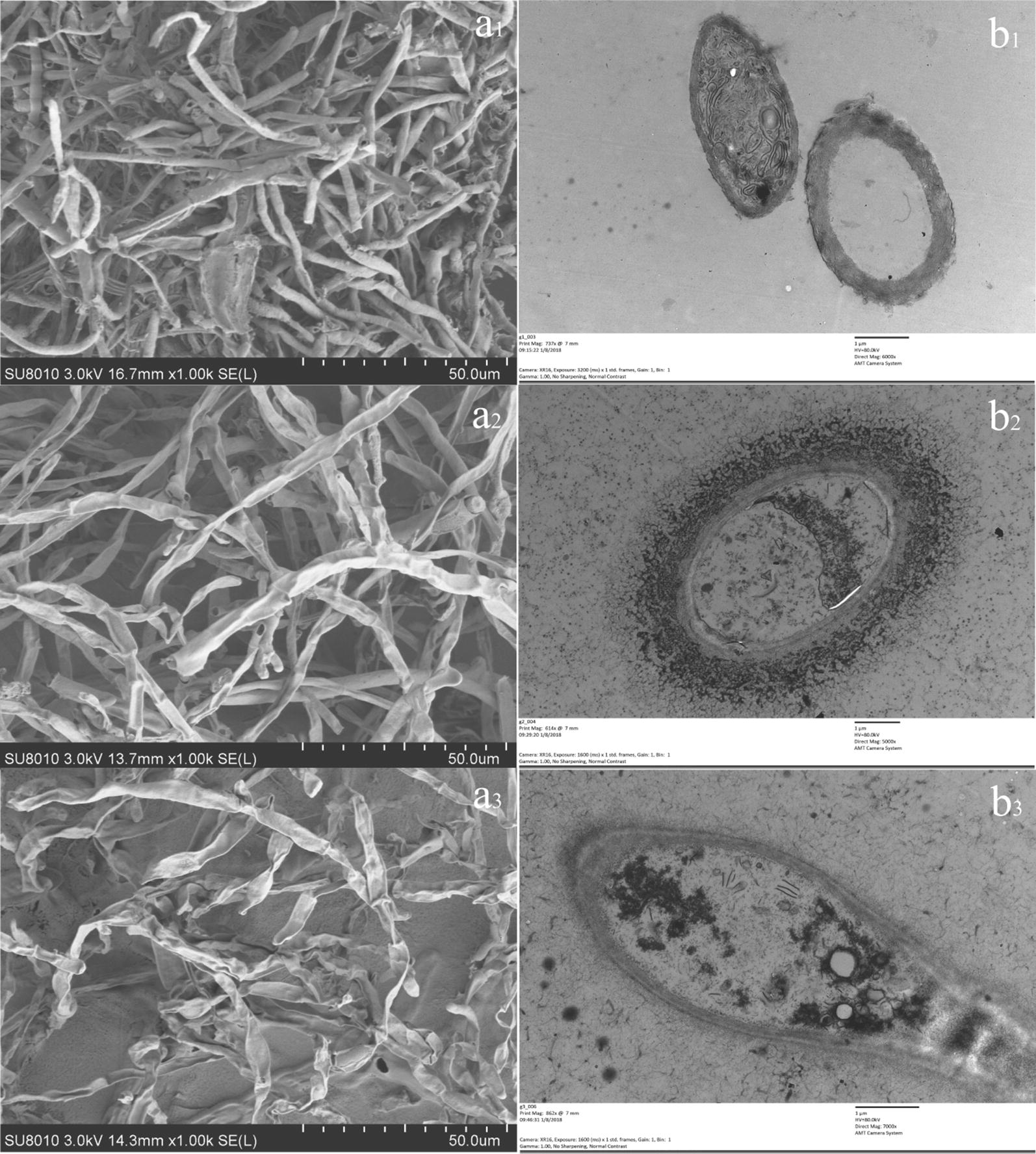
Electron microscope detection of *C. gloeosporioides*. The images on the left side of the figure show scanning electron micrographs of *C. gloeosporioides* and the images on the right side show transmission electron micrographs. **a1** and **b1** are the images from the blank control group, **a2** and **b2** are images from the 2,4-di-*tert*-butylphenol treatment, **a3** and **b3** are the images from the CF-3 24hFB treatments

The effect of *B. subtilis* CF-3 VOCs on the mycelial morphology of *C. gloeosporioides* is showed in Fig. 2. The scanning electron microscopy showed that the hyphae in the blank group were intact and their surface was smooth and plump (a_1_); after treatment with 2,4-di-*tert*-butylphenol, the hyphae shrunk and ruptured (a_2_); after treatment with CF-3 24hFB, the hyphae shrunk and twisted, leading to a sinuous or wrinkled surface (a_3_). The transmission electron microscopy showed that the hyphae of the blank group exhibited a typical fungal ultrastructure (b_1_); after treatment with 2,4-di-*tert*-butylphenol, the protoplast membrane was broken, and the cell wall was separated, and the intracellular components were severely destroyed, forming a shell (b_2_); after treatment with CF-3 24hFB, the cell organelles began to shrink (b_3_). These results showed that the VOCs fumigation treatment can cause the hyphae of *C. gloeosporioides* to shrink and rupture, cell organelles to shrink and form vacuoles, and cell wall to break, thereby inhibiting the growth of *C. gloeosporioides*.

### Effect of VOCs on fatty acid content in *C. gloeosporioides* cell membrane

The results of the GC–MS analysis showed that the blank control group contains more long-chain unsaturated fatty acids and a small amount of saturated fatty acids. The relative content of *cis*-linoleic acid decreased and that of *trans*-linoleic acid, oleic acid, palmitic acid, and stearic acid increased after 2,4 di-*tert*-butylphenol and CF-3 24hFB treatment (Table 1), indicating that the fatty acid unsaturation in the cell membrane decreased and the membrane fluidity weakened, resulting in the leakage of contents.

**Table 1:** Fatty acid content of cell wall of *C. gloeosporioides*

Fatty acid	Treatment		
	No treatment	2,4-Di-*tert*-butylphenol	CF-3 24h FB
Palmitic acid C16:0 (%)	22.16 ± 3.28 b	21.24 ± 3.09 b	27.87 ± 1.57 a
Stearic acid C18:0 (%)	0 c	8.17 ± 1.84 b	16.93 ± 2.51 a
9-Hexadecenoic acid C16:1 (%)	0 c	1.23 ± 0.27 a	0.53 ± 0.08 b
16-Heptadecenoic acid C17:1 (%)	14.22 ± 1.52 a	0 b	0 b
Oleic acid C18:1 (%)	0 c	36.97 ± 4.21 a	28.63 ± 2.48 b
Trans 9-octadecenoic acid C18:1 (%)	0 c	1.19 ± 0.18 a	0.62 ± 0.07 b
Linoleic acid C18:2 (%)	29.67 ± 3.03 a	0 c	3.41 ± 0.95 b
Trans-linoleic acid C18:2 (%)	20.90 ± 1.67 b	31.20 ± 2.94 a	22.01 ± 1.23 b
8,11,14-Docosic acid C22:3 (%)	13.05 ± 2.16 a	0 b	0 b
Unsaturated/saturated	3.51 ± 0.24 a	2.40 ± 0.29 b	1.23 ± 0.15 c

The mean and standard error in the table were taken from three replicates, and the different letters indicated that the different treatments of the same strain had significant differences (*P *<* 0.05*)

### Effects of VOCs on the content of ergosterol in the *C. gloeosporioides* cell wall

In this test, the standard curve of ergosterol was y = 20.563x + 0.3462, with a correlation coefficient of 0.9995 (Table 2), which showed a good correlation. In the blank control group, the content of ergosterol in the cell wall treated with 2,4-di-*tert*-butylphenol and CF-3 24hFB was significantly reduced (*P *<* 0.05*). This showed that the treatment with VOCs reduced the content of ergosterol in the cell wall of *C. gloeosporioides* and affected the integrity of the fungal cell wall, thus weakening the cell membrane flow and material transport, affecting the viability of fungal cells.

**Table 2:** Content of ergosterol in *C. gloeosporioides*

	Treatment	Peak area ratio	Ergosterol content (mg/g)
Electron microscope	No treatment	5.9118 ± 0.1523	3.2479 ± 0.0889 a
	2,4-Di-*tert*-butylphenol	3.4303 ± 0.1689	1.7998 ± 0.0986 b
	CF-3 24 hFB	3.0325 ± 0.1153	1.5677 ± 0.0673 b

The mean and standard error in the table were taken from three replicates, and the different letters indicated that the different treatments of the same strain had significant differences (*P *<* 0.05*)

### Effects of CF-3 VOCs on related enzyme activities and activity indexes

#### Pathogenic enzymes

**Fig. 3:**
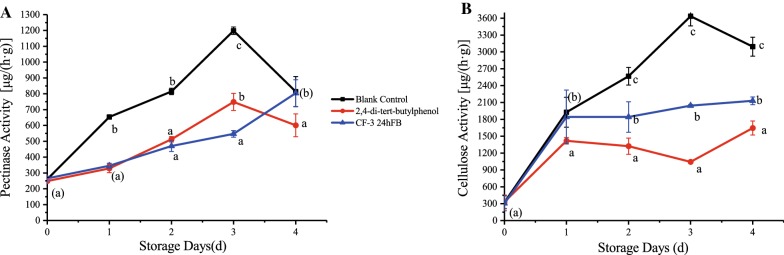
Influence of different treatments on pectinase (**A**) and cellulase (**B**) activity in vivo during storage. The mean and standard error in the figure were taken from three replicates, and different letters indicate that different treatments on the same storage days were significantly different (*P *<* 0.05*)

As shown in Fig. 3, the polygalacturonase activity in litchi fruits of the three groups increased with increasing storage days, but the increasing rate and enzyme activity of the blank group were much higher than that of the two treatment groups, indicating that 2,4-di-*tert*-butylphenol and CF-3 24hFB can inhibit the increase in the activity of polygalacturonase. The cellulose activity in the blank group showed a significant upward trend with the increase in the number of storage days (Fig. 3B), while the activity of the two treatment groups leveled off after d2. The two treatment groups showed a slight increase on d4, but the enzyme activity was significantly lower than that of the blank group, indicating that 2,4-di-*tert*-butylphenol and CF-3 24hFB treatment can significantly inhibit the increase in cellulose activity.

#### Active oxygen

**Fig. 4:**
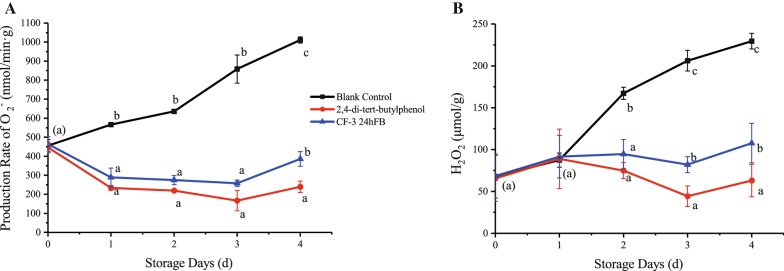
Influence of different treatments on the production rate of superoxide anion (**A**) and the amount of hydrogen peroxide (**B**) produced in vivo during storage. The mean and standard errors in the figure were taken from three replicates, and different letters indicate that different treatments in the same storage days were significantly different (*P *<* 0.05*)

The production rate of superoxide anion and hydrogen peroxide content during the storage of litchi fruits are shown in Fig. 4. During the whole storage process, the production rate of superoxide anion in the two treatment groups showed a significantly decreasing trend in contrast to the blank group. The production rate of the superoxide anion in the 2,4-di-*tert*-butylphenol treatment group decreased continuously until d3, and increased slightly on d4, but was lower than the other groups, indicating that 2,4-di-*tert*-butylphenol can induce oxidation resistance, thereby inhibiting the production rate of superoxide anion in litchi fruit. For hydrogen peroxide, the content in the treatment groups decreased significantly after 2 days, and the content on d3 reached its lowest level. In general, the hydrogen peroxide content in the 2,4-di-*tert*-butylphenol treatment group was significantly lower than the other two groups.

#### Degree of cell damage

**Fig. 5:**
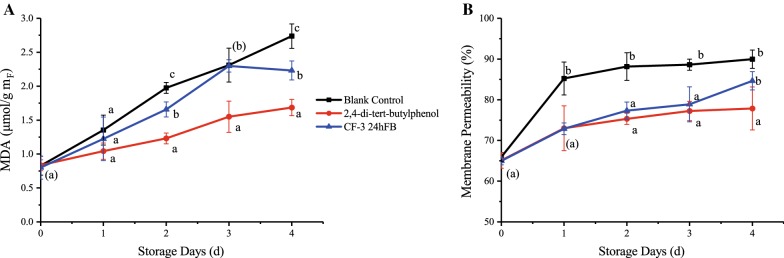
Influence of different treatments on the amount of MDA (**A**) and membrane permeability (**B**) in vivo during storage. The mean and standard errors in the figure were taken from three replicates, and different letters indicate that different treatments in the same storage days were significantly different (*P *<* 0.05*) (*MDA* malondialdehyde)

The changes in MDA content and cell membrane permeability during storage are shown in Fig. 5. During the whole storage process, the MDA content in the litchi fruits of the two treatment groups increased with the storage time, but the inhibiting effects of the 2,4-di-*tert*-butylphenol and CF-3 24hFB treatments were significantly better than the control treatment. During the storage process, the cell membrane permeability increased continuously, indicating that the fruit cells became more and more severe with the increase of storage days. Generally, the MDA content and cell membrane permeability in the treatment groups were the lowest, indicating that the 2,4-di-*tert*-butylphenol and CF-3 24hFB treatments can effectively prevent cell membrane lipid peroxidation.

#### Antioxidant enzymes

**Fig. 6:**
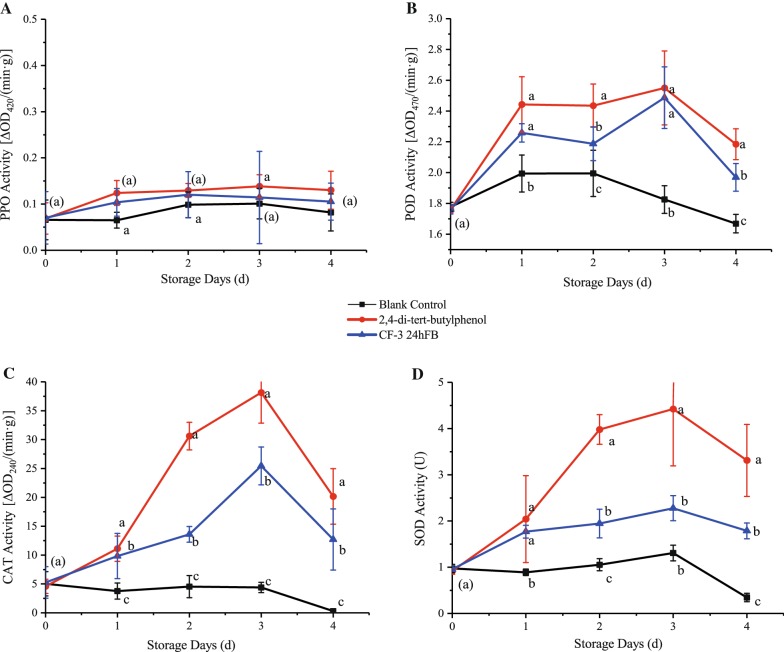
Influence of different treatments on POD (**A**), PPO (**B**), CAT (**C**) and SOD (**D**) activity in vivo during storage. The mean and standard errors in the figure were taken from three replicates, and different letters indicate that different treatments in the same storage days were significantly different (*P *<* 0.05*) (*POD* peroxidase, *PPO* polyphenol oxidase, *CAT* catalase, *SOD* superoxide dismutase)

The activities of four antioxidant enzymes (PPO, POD, CAT, and SOD) showed first an increasing trend and then a decreasing trend during storage (Fig. 6). Except PPO, the activities of the three enzymes were significantly higher in the two treatment groups than in the blank control group, while the PPO activities were slightly higher in the treatment groups than in the blank group, indicating that both treatments can induce the production of antioxidant enzymes in the fruit to remove excess hydrogen peroxide and free radicals from the fruit. The activities in the 2,4-di-*tert*-butylphenol treatment group were higher than those in the CF-3 24hFB treatment group, and both reached the peak on d3, indicating that 2,4-di-*tert*-butylphenol can effectively induce fruit oxidation resistance to prevent oxidative damage. This result corresponds to the result of the lowest MDA content and cell membrane permeability in the 2,4-di-*tert*-butylphenol treatment group.

#### Disease-resistant enzymes

**Fig. 7:**
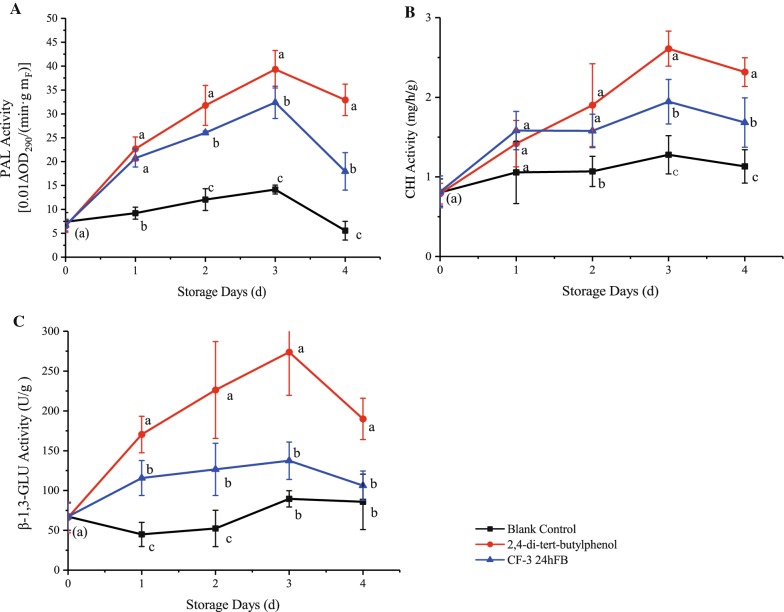
Influence of different treatments on PAL (**A**), CHI (**B**) and β-1,3-GLU (**C**) activity in vivo during storage. The mean and standard errors in the figure were taken from three replicates, and different letters indicate that different treatments performed on the same storage days were significantly different (*P *<* 0.05*) (*PAL* phenylalanine ammonia lyase, *CHI* chitinase, *β-1,3-GLU* β-1,3-glucanase)

The activities of the three enzymes related to plant disease resistance (PAL, CHI, and β-1,3-GLU) showed an increasing trend followed by a decreasing trend during storage (Fig. 7), and the enzyme activities in the two treatment groups were all higher than the blank control group, indicating that both treatments can induce fruit disease resistance. The enzyme activities in the 2,4-di-*tert*-butylphenol treatment group were significantly higher than those in the CF-3 24hFB treatment group, and both reached the peak on d3, indicating that 2,4-di-*tert*-butylphenol can effectively induce plant disease resistance and resist fungal attack.

## Discussion

In general, the effect of 2,4-di-*tert*-butylphenol is better than that of CF-3 24hFB. 2,4-di-*tert*-butylphenol is one of the components isolated from CF-3 24hFB by headspace SPME–GC–MS (HS–SPME–GC–MS) in our previous experiments. The results of earlier bacteriostatic experiments showed that many compounds in CF-3 24hFB had no bacteriostatic effect, while 2,4-di-*tert*-butylphenol had the best bacteriostatic effect on *C. gloeosporioides*. Mi-Ae et al. (2006) found that 2,4-di-*tert*-butylphenol has good antioxidant activity and has many great effects in scavenging free radicals. Sang and Kim (2012) also isolated 2,4-di-*tert*-butylphenol from VOCs produced by bacterial strain (GSE09) antagonistic to *Phytophthora capsici* on pepper and proved to be bacteriostatic, which was the same as our previous bacteriostatic results. Therefore, 2,4-di-*tert*-butylphenol was selected as the positive control to compare the antibacterial effect horizontally. In this experiment, CF-3 24hFB had a significant inhibitory effect on *C. gloeosporioides*, and the antibacterial effect of the 2,4-di-*tert*-butylphenol treatment group was better than that of the CF-3 24hFB treatment group.

The cell wall and membrane not only maintain the shape of the cell, increasing the mechanical strength of the cell, but also control a part of the physical and information transport while bearing the swell pressure generated by the protoplasts, and provides certain disease resistance. The scanning and transmission electron microscopies showed that after fumigating with 2,4 di-*tert*-butylphenol and CF-3 24hFB, the hyphae were shrunk, twisted, and broken, resulting in a striated or wrinkled surface. In addition, the intracellular components were seriously damaged, the organelles began to shrink, the plasmalemma ruptured, and the cell wall contents completely leaked, forming an empty shell. It can be seen that 2,4 di-*tert*-butylphenol and VOCs in CF-3 24hFB can act on the cell wall and cell membrane system of fungi, destroying their basic functions.

The lipid composition of the fungal cell membrane and its contents are also an important parameter of the cell membrane. Lipids have many important functions, including increasing cell membrane stability, regulating cell membrane fluidity, and reducing the permeability of water-soluble substances (Heaton and Randall 2011). Regarding the relationship between the content of unsaturated fatty acids in fungi and cell membrane integrity, Cronan (2015) found that if the cell membrane lacked unsaturated fatty acids, it would cause loss of content and dissolution. Bayer et al. (2000) found that if the saturated fatty acid content in the cell membrane was at high, it would lead to an increase in cell membrane stiffness and a decrease in membrane fluidity, which might easily cause cell rupture. In this experiment, the degree of unsaturation of VOC-treated fungal cells decreased to varying extents, likely reflecting a VOC-mediated decrease in fungal cell membrane fluidity, which led to cell rupture and leakage of contents.

In addition, ergosterol is present in various fungi, and it is a major component of the cell wall of filamentous fungi. It plays an essential role in the growth and function of cells. In addition to controlling the fluidity, asymmetry, and integrity of cell membranes, ergosterol is helpful for the normal activities of enzymes in cell wall. Pinto et al. (2011) reported that 1,2-dihydroxy xanthone had high antibacterial activity as well as reduced the ergosterol contents of *Trichophyton mentagrophytes*, *Aspergillus fumigatus*, *Candida albicans*, and *Cryptococcus neoformans*. This was consistent with the bacteriostatic effect of *B. subtilis* CF-3 VOCs observed in this study. In this experiment, the content of ergosterol in the cell walls of the VOC-treated fungus decreased to varying extents, which could reflect that VOCs destroyed the fungal cell wall and interfered with the normal function of the cell membrane.

Pectinase and cellulase are cell wall degrading enzymes produced by pathogenic bacteria, which are one of the factors causing fruit disease (Amadioha 2010). 2,4-di-*tert*-butylphenol and VOCs can inhibit the activity of the two pathogenic enzymes, thereby protecting the pectin and cellulose components of the litchi fruits from the two pathogenic enzymes.

Superoxide anion and hydrogen peroxide will be produced in the fruit body. Excessive superoxide anion and hydrogen peroxide can induce the peroxidation of unsaturated fatty acids in membrane lipids, produce lipid free radicals, and further induce the peroxidation of membrane lipids, leading to increased membrane permeability and cell damage or death (Macarisin et al. 2010). MDA is one of the main products of membrane lipid peroxidation, and its content can be used as an indicator of lipid peroxidation, reflecting the degree of membrane lipid peroxidation. At the same time, when the cell membrane of the fruit and vegetable tissues is damaged, electrolyte extravasation in the cell membrane will increase the electrical conductivity, which can reflect the degree of injury to the fruit and vegetable (Liang 2008). 2,4-di-*tert*-butylphenol and VOCs can inhibit the production rate of superoxide anion in litchi fruit, prevent the occurrence of membrane lipid peroxidation and remove excess hydrogen peroxide from the fruit.

POD, PPO, CAT and SOD are four typical antioxidant enzymes in fruits. The enzyme activity of these four enzymes can reflect the ability of the plant to remove excess oxygen free radicals and its antioxidant capacity (Zheng et al. 2007). PAL, CHI and β-1,3-GLU are typical disease-resistant enzymes in fruits. These three enzymes are closely related to the resistance to stress and disease of plants, and play an important role in the normal growth and development of plants and the resistance to pathogen invasion (Fortunato et al. 2015). 2,4-di-*tert*-butylphenol and VOCs can induce the production of antioxidant enzymes and disease-resistant enzymes in the fruit, effectively induce plant disease resistance and resist fungal attack.

In this study, the physical and flat-panel experiments show that VOCs produced by *B. subtilis* CF-3 can prevent the decay of litchi fruits infected by *C. gloeosporioides* and can significantly inhibit the spore germination and growth of pathogenic bacteria. Meanwhile, it was further proved that VOCs can destroy the structure of fungal cells and induce post-harvest disease resistance in litchi fruits.

## Data Availability

The data supporting the conclusions of this article are included within the article. Data and materials can also be requested from the corresponding author.
